# Supplementary figures and images for: Lassa viral dynamics in non-human primates treated with favipiravir or ribavirin
Source: PLoS Comput Biol. 2021 Jan 7;17(1):e1008535. doi: 10.1371/journal.pcbi.1008535 (PMC7817048; doi:10.1371/journal.pcbi.1008535)

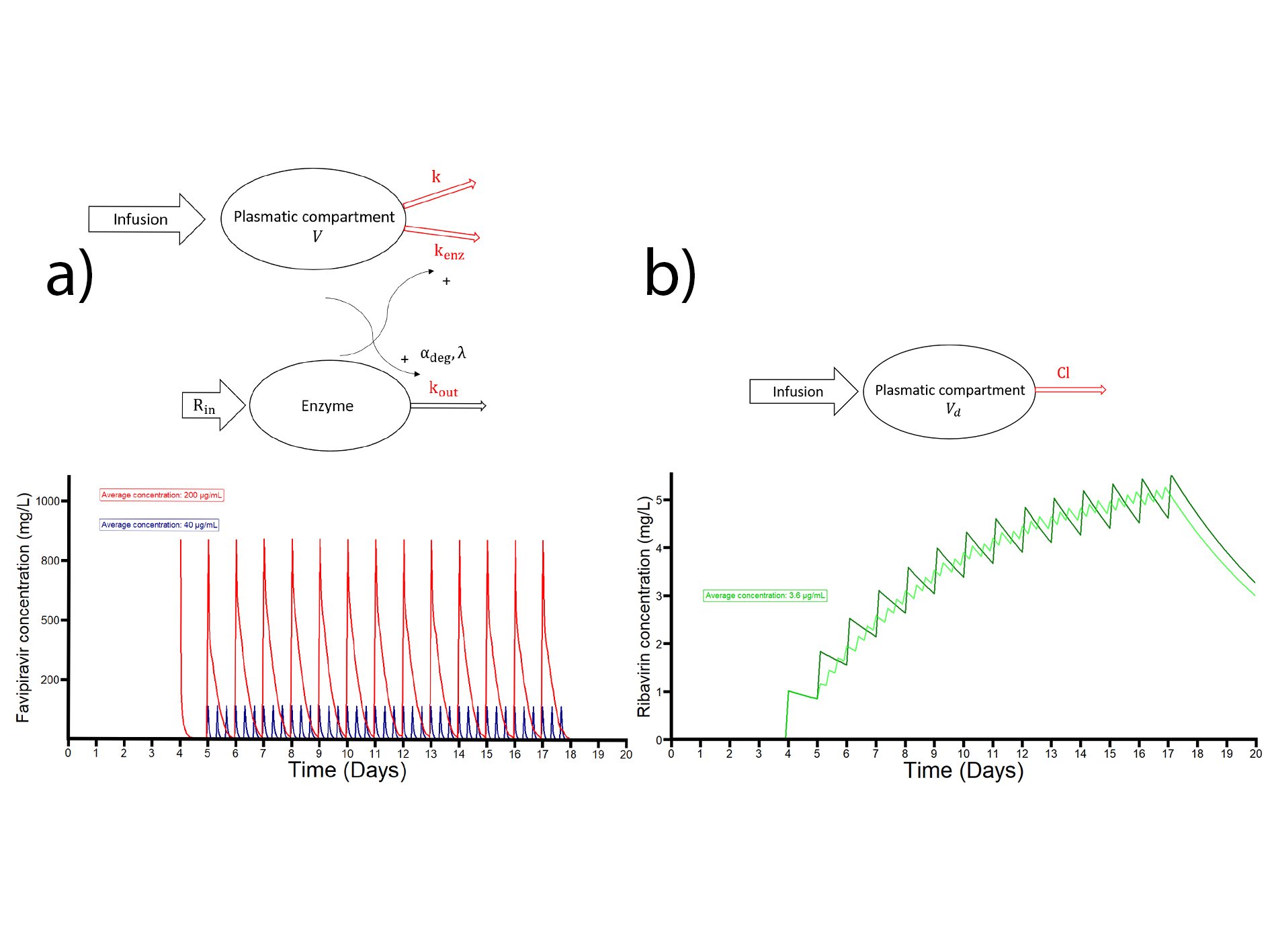

Supplement: S1 Fig — a). Favipiravir model. b). Ribavirin model. (TIF) [file pcbi.1008535.s001.tif]

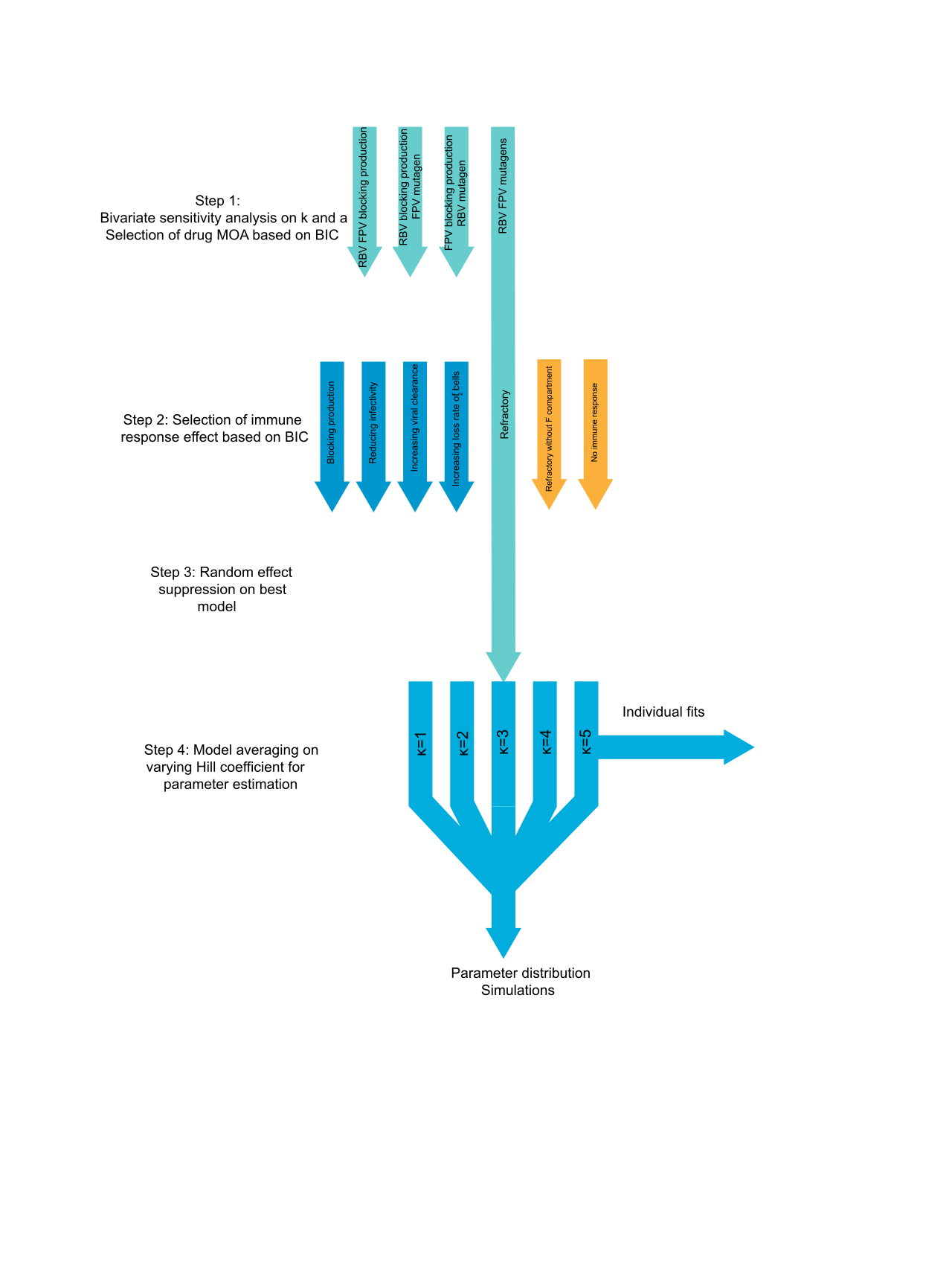

Supplement: S2 Fig — Steps of model selection. (TIFF) [file pcbi.1008535.s002.tiff]

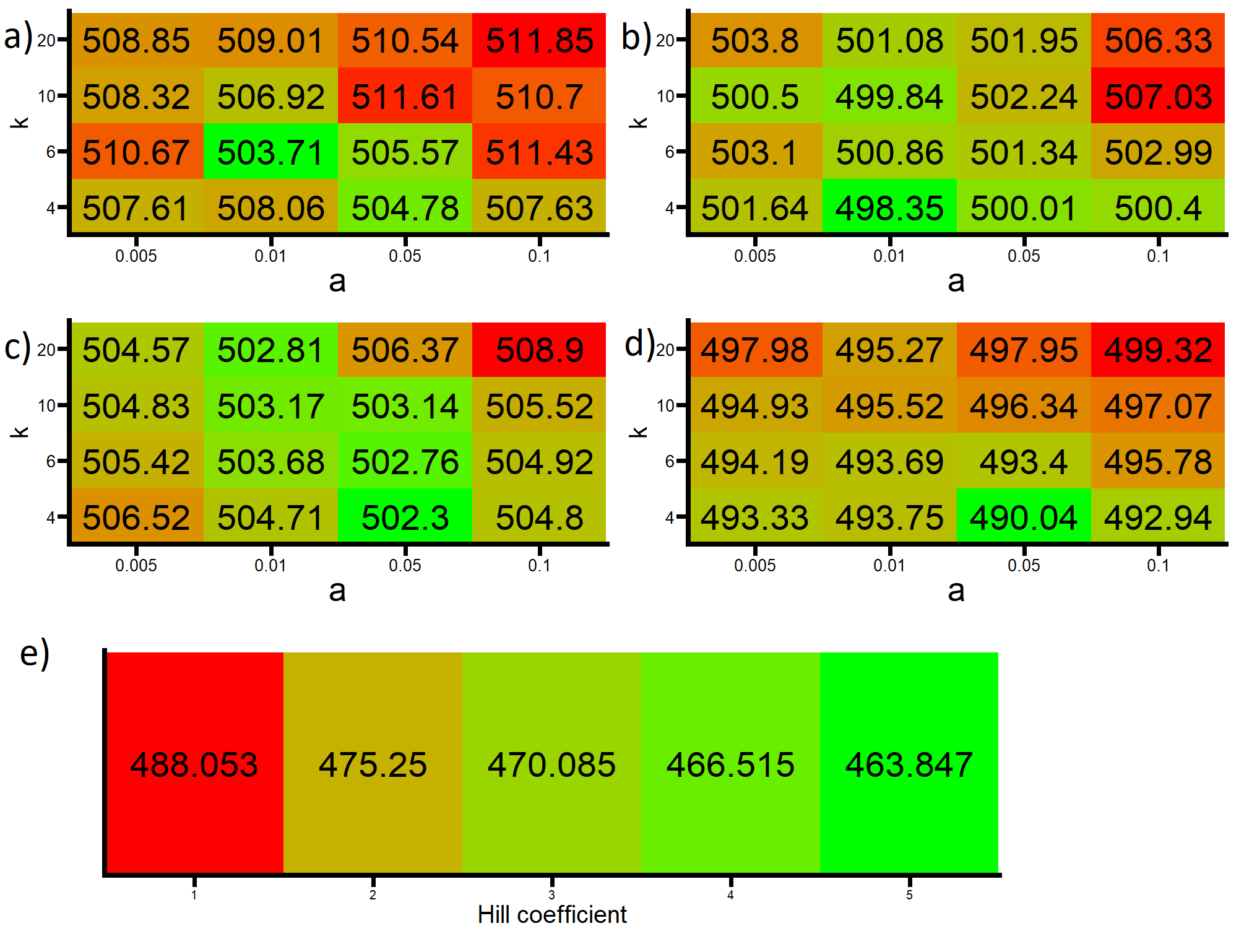

Supplement: S3 Fig — A-D) Step 1. A) FPV and RBV blocking production. B) FPV mutagen, RBV blocking production. C) RBV mutagen, FPV blocking production. D) FPV and RBV mutagens. E) Step 2-3. Mutagenesis model after random effect selection. (TIF) [file pcbi.1008535.s003.tif]

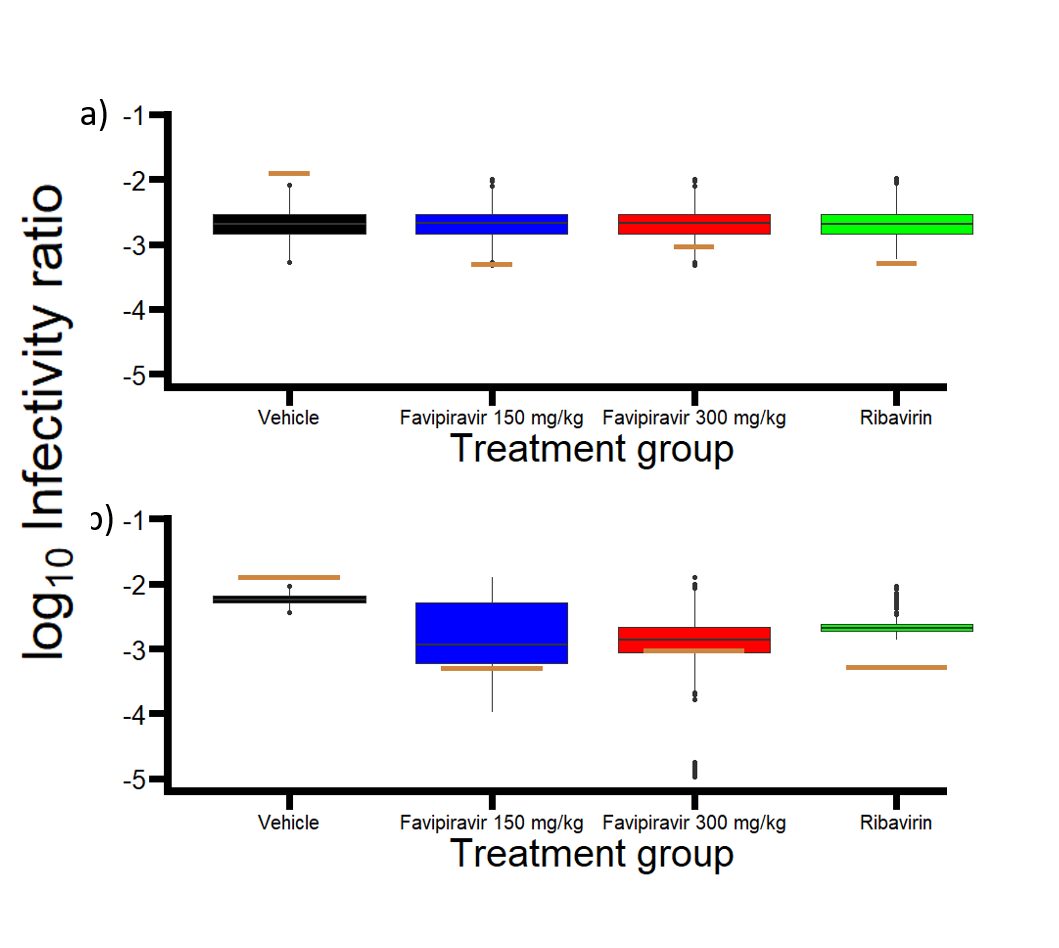

Supplement: S4 Fig — Top: Production blockage model. Bottom: Mutagenesis model. Yellow bars represent observed medians of each group. (TIF) [file pcbi.1008535.s004.tif]

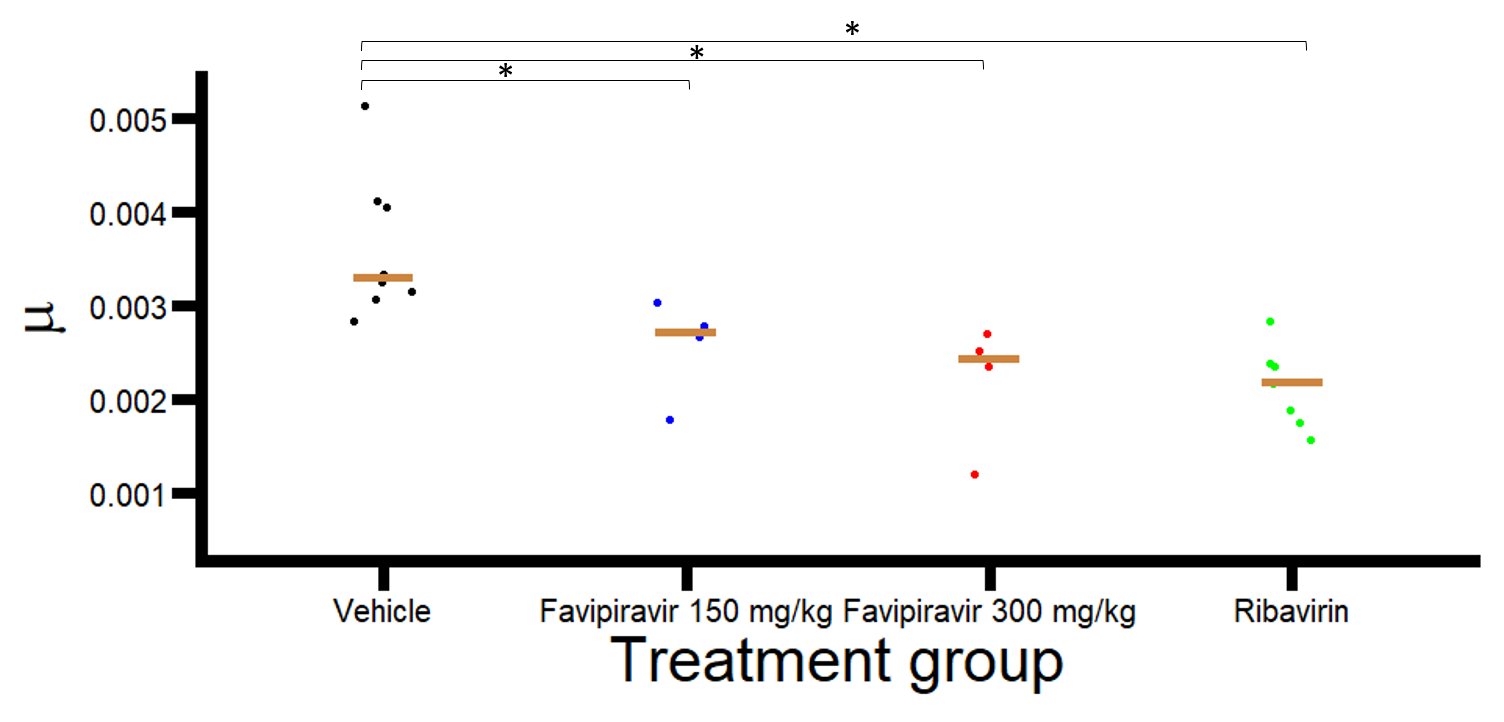

Supplement: S5 Fig — EBEs were obtained using the production blockage model. * = p < 0.05. Horizontal lines represent medians by group. (TIF) [file pcbi.1008535.s005.tif]

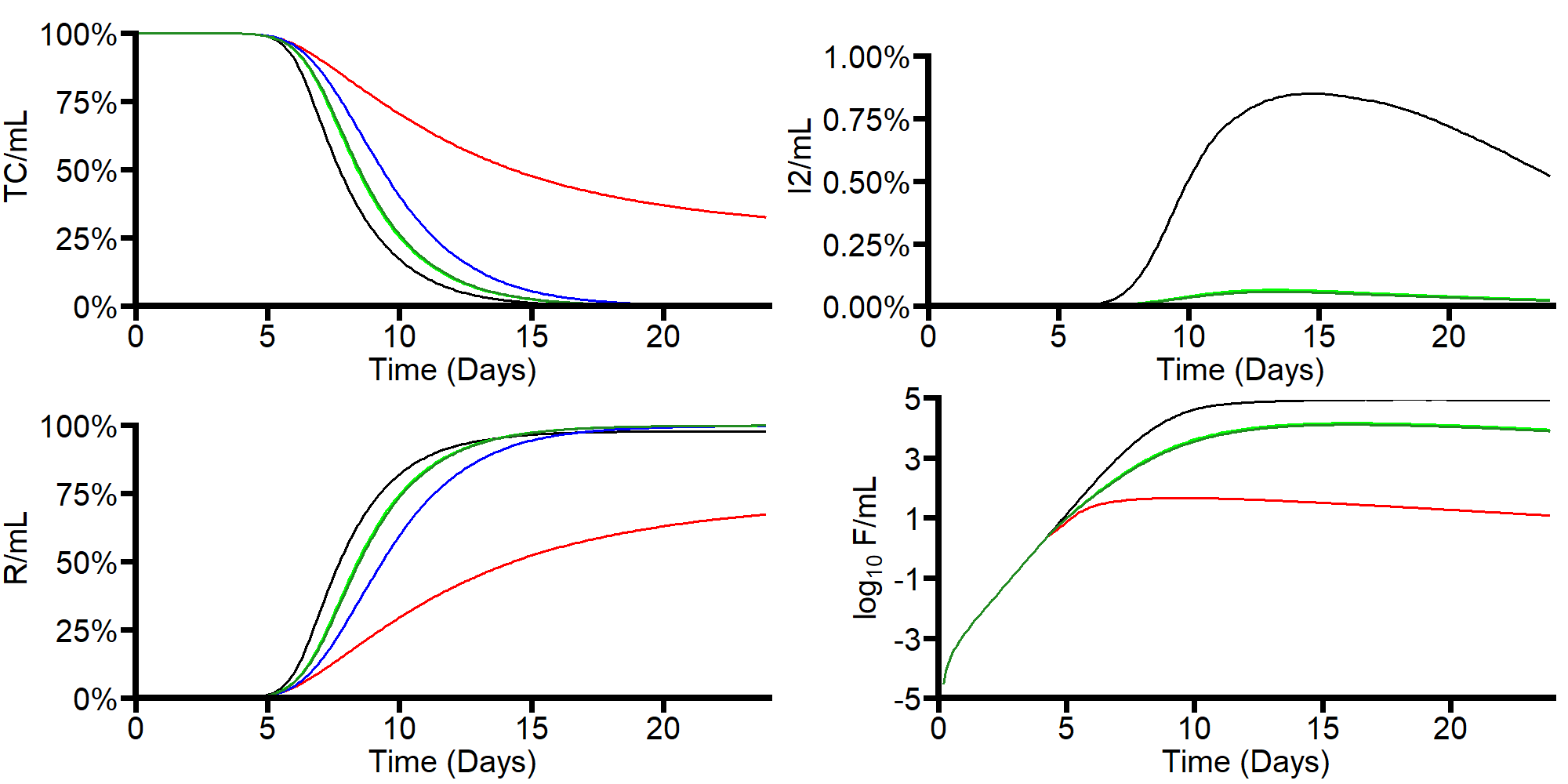

Supplement: S6 Fig — Black curve: animals receiving vehicle treatment; green: animals treated with RBV; blue: animals treated with FPV 150 mg/kg/day; red: animals treated with FPV 300 mg/kg/day. (TIF) [file pcbi.1008535.s006.tif]

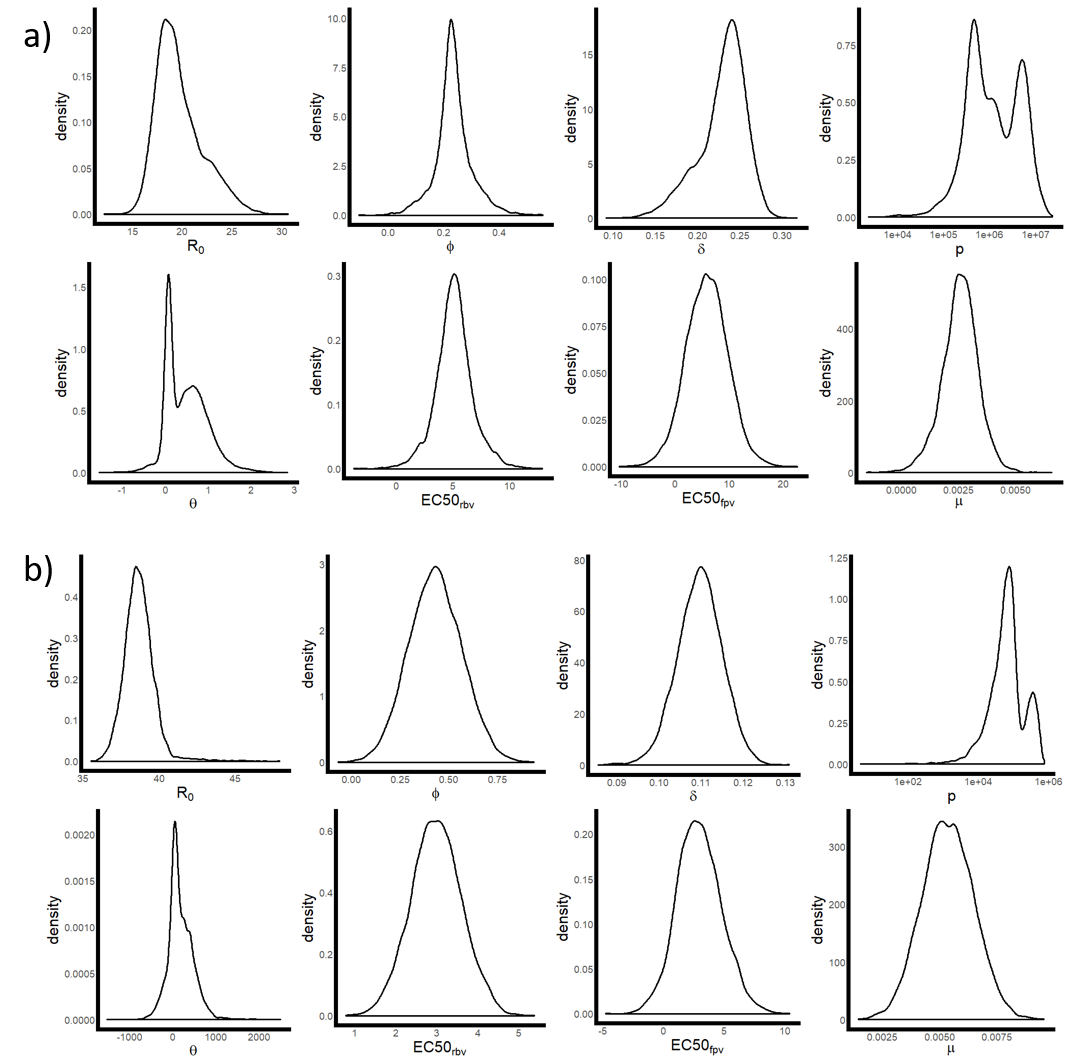

Supplement: S7 Fig — Top: Production blockage model. Bottom: mutagenesis model. (TIF) [file pcbi.1008535.s007.tif]
