# Supplementary material for: Lassa viral dynamics in non-human primates treated with favipiravir or ribavirin
Source: PLoS Comput Biol. 2021 Jan 7;17(1):e1008535. doi: 10.1371/journal.pcbi.1008535 (PMC7817048; doi:10.1371/journal.pcbi.1008535)
Supplement: S1 Table — Values are estimated population values. (PDF) [file pcbi.1008535.s009.pdf]

Drug	Parameter	Value
Favipiravir	V ($L.kg^{-1}$)	0.359
	k (h^{-1})	0.0654
	k_{enz} (h^{-1})	2.85
	α_{deg} ($mg^{-1}L$)	0.179
	k_{out} (h^{-1})	0.024
	λ (h^{-1})	0.00155
Ribavirin	V_d ($L.kg^{-1}$)	29.4
	Cl ($ml/h/kg$)	224

Table S1. Pharmacokinetic parameters of favipiravir and ribavirin
