# Supplementary material for: Lassa viral dynamics in non-human primates treated with favipiravir or ribavirin
Source: PLoS Comput Biol. 2021 Jan 7;17(1):e1008535. doi: 10.1371/journal.pcbi.1008535 (PMC7817048; doi:10.1371/journal.pcbi.1008535)
Supplement: S2 Table — FPV and RBV mutagen agents, κ = 1. (PDF) [file pcbi.1008535.s010.pdf]

Immune response	Cytotoxicity	Blocking infection	Blocking production	Increasing viral clearance	Refractory
**BIC**	511.45	499.89	506.95	506.20	490.70

Table S2. BIC Comparison between modes of action of the immune response. FPV and RBV mutagen agents, κ=1
