# Supplementary material for: Lassa viral dynamics in non-human primates treated with favipiravir or ribavirin
Source: PLoS Comput Biol. 2021 Jan 7;17(1):e1008535. doi: 10.1371/journal.pcbi.1008535 (PMC7817048; doi:10.1371/journal.pcbi.1008535)
Supplement: S3 Table — Refractory model with FPV and RBV mutagen agents, κ = 1. (PDF) [file pcbi.1008535.s011.pdf]

**Immune response**	**Refractory with density dependent term**	**No immune response**	**Refractory without F compartment**
**BIC**	490.70	507.33	496.63

Table S3. F compartment assessment. Refractory model with FPV and RBV mutagen agents, κ=1
