# Supplementary material for: Lassa viral dynamics in non-human primates treated with favipiravir or ribavirin
Source: PLoS Comput Biol. 2021 Jan 7;17(1):e1008535. doi: 10.1371/journal.pcbi.1008535 (PMC7817048; doi:10.1371/journal.pcbi.1008535)
Supplement: S1 Text — Equations for the pharmacokinetic model of favipiravir described in the litterature. (PDF) [file pcbi.1008535.s012.pdf]

# Favipiravir pharmacokinetics

Favipiravir pharmacokinetic model is driven by the following equations:

$$\frac{dA_c}{dt} = -kA_c - k_{enz}A_eA_c \tag{1}$$

$$\frac{dA_e}{dt} = R_{in} - k_{out}(1 + C_c\alpha_{deg}e^{\lambda t})A_e \tag{2}$$

$$\frac{dR_{in}}{dt} = k_{out}A_{e0} \tag{3}$$

$$\frac{dC_c}{dt} = \frac{A_c}{V} \tag{4}$$

With $A_c$ the amount of favipiravir in the central compartment, $C_c$ the FPV plasmatic concentration, $A_e$ the enzymatic activity level, $k$ the first-order elimination rate, $k_{enz}$ the enzyme-dependent first-order elimination rate, $k_{out}$ the enzyme elimination rate, $R_{in}$ is the zero-order enzyme synthesis rate, $\alpha_{deg}$ the linear effect of the favipiravir concentration on the enzyme elimination rate, $V$ the volume of distribution of FPV and $\lambda$ the rate at which enzyme elimination decreases, with $A_{e0}$ set as 1 at the beginning of the infusion.
