# Supplementary material for: Lassa viral dynamics in non-human primates treated with favipiravir or ribavirin
Source: PLoS Comput Biol. 2021 Jan 7;17(1):e1008535. doi: 10.1371/journal.pcbi.1008535 (PMC7817048; doi:10.1371/journal.pcbi.1008535)
Supplement: S2 Text — Equations of models tested for model selection. (PDF) [file pcbi.1008535.s013.pdf]

Immune response models tested

The different alternative models can be written as:

$$\text{Infectivity}\begin{cases} \frac{dT}{dt} = -\beta(1 - \phi T\frac{F}{F+\theta})V_iT & \text{(1a)} \\ \frac{dI_1}{dt} = \beta(1 - \phi T\frac{F}{F+\theta})V_iT - kI_1 & \text{(1b)} \end{cases}$$

$$\text{Cytotoxicity} \quad \frac{dI_2}{dt} = kI_1 - \delta I_2 - \phi I_2\frac{F}{F+\theta} \tag{2}$$

$$\text{Viral production}\begin{cases} \frac{dV_{ni}}{dt} = p(1 - \phi\frac{F}{F+\theta})(1-\mu)I_2 - cV_{ni} & \text{(3a)} \\ \frac{dV_i}{dt} = p(1 - \phi\frac{F}{F+\theta})\mu I_2 - cV_i & \text{(3b)} \end{cases}$$

$$\text{Viral clearance}\begin{cases} \frac{dV_{ni}}{dt} = p(1-\mu)I_2 - cV_{ni} - \phi V_{ni}\frac{F}{F+\theta} & \text{(4a)} \\ \frac{dV_i}{dt} = p\mu I_2 - cV_i - \phi V_i\frac{F}{F+\theta} & \text{(4b)} \end{cases}$$

$$\text{Other models tested}\begin{cases} \frac{dT}{dt} = -\beta V_iT \quad \text{if } \theta \text{ is set to } \infty & \text{(5a)} \\ \frac{dT}{dt} = -\beta V_iT - \phi T \quad \text{if } \theta \text{ is set to } 0 & \text{(5b)} \end{cases}$$
