# Supplementary material for: Lassa viral dynamics in non-human primates treated with favipiravir or ribavirin
Source: PLoS Comput Biol. 2021 Jan 7;17(1):e1008535. doi: 10.1371/journal.pcbi.1008535 (PMC7817048; doi:10.1371/journal.pcbi.1008535)
Supplement: Attachment — Submitted filename: Response to reviewers_Lassa_final.docx [file pcbi.1008535.s014.docx]

**Lassa viral dynamics in non-human primates treated with favipiravir or ribavirin**

**Response to reviewers:** Guillaume Lingas, Kyle Rosenke, David Safronetz, Jérémie Guedj

Dear Editor,

We would like to thank you and the two anonymous reviewers for their interest and thorough reviews, which greatly improved the manuscript. Please find below our rebuttal letter. The references are listed at the end of the document with internal numbering.

**Reviewer #1: The paper describes the first mathematical model of Lassa virus infection and uses NHP data to determine the roles of favipiravir and ribavirin in reducing the virus load and inducing viral cure. While I believe the study is important and has translational potential, the model development, the data fitting, and the model results need a more clear presentation. I will detail that below:**

**- It is very difficult to determine which results are based on the data and which are based on the model. This is especially true since the authors use the word dynamic to describe the mathematical model but also Table 1 which I believe is based on data. Same question regarding figures 1 and 2, are they based on data? Clarify.**

We now use the terminology “viral kinetics” to refer to raw data and “viral dynamics” to refer to modeling results and predictions. Accordingly, we changed the titles of subsection 1 to “descriptive analysis of viral kinetics”, Table 1 to “Description of viral kinetics”, Figure 1 to “Observed survival according to treatment received”

**- The mathematical model is quite confusing. Why are you using X for multiplication in some terms but not in others? Why is there an influx a*D/vol*e^(-a*c_t)t in the infectious virus population? What is D? Why is a accounted twice in that term? Define all these parameters. You call vol=V in the paper, chose one notation.**

We thank the reviewer for pointing out our typos and we modified the text per your comment.

We also clarified the role of D in the introduction of the model and we refer to Best et al. (1):

“As an initial infectious viral load is required to model the start of the infection, we used a model proposed by Best et al. to model the progressive arrival of the viral inoculum to the infection site after intramuscular injection. In this model the initial inoculum dose, D (equal to 10^4^ TCID_50_), is diluted into the plasmatic volume, vol (equal to 300 mL in macaques) and is transported to the site of infection with rate *a*. During the transport, the virus can also be eliminated, with a clearance rate $c_{t}$ .” Line 116

**-Eclipse phase is I1 not I2.**

We thank the reviewer for pointing out the typo, that was corrected.

**-presentation of the density dependent terms needs to be closer to the standard. Define Phi and name k=Hill coefficient. Talk about what having a higher k means in terms of cooperative effects.**

We added definitions of phi and Hill coefficients as well as a more precise definition of the immune response effect lines 110-115:

“The effect of the immune response is therefore represented by the term $\phi\frac{F}{F+\theta}$. In this model, the presence of viral antigen stimulates the innate immune response, represented by the compartment $F$, which activates and renders cells refractory to infection, at a maximal rate noted $\phi,$ while $\theta$ represents the concentration of $F$ required to achieve 50% of the maximal rate. […] In the case of FPV, for which two different doses were used, we also considered the possibility of a sigmoidicity parameter to model the concentration effect relationship (Hill coefficient, noted $\kappa$), ranging from 1 to 5 (close to an "on-off" effect).”

**- The PK model in the figure S1 implies a more complicated form. Have you fitted it to PK data? Explain more what it does, how did you obtain the magnitude and the decay, and move it to the main manuscript, especially since it forms the basis for functions C.**

We did not have PK measurements in this study, and we relied on a model that has been developed at the same daily dose in the macaque species in our previous experiments on Ebola virus (2). Thus, the PK parameters were fixed in all animals to the mean values found in (2), as stated in line 137: ‘Given the absence of pharmacokinetic data in the experiment, we fixed all individual parameters to the mean values found in the literature (2, 3) (see S1 Table and predictions in S1 Fig).”

As no PK data were collected in this work, we chose to not present the model in the main text. However, we ensure full reproducibility of our results and we added the equations and the parameter values in the Supplementary material (S1 Figure Table, S1 Figure and Supplementary Eqs. 1-4 in Supplementary methods).

“[…] complex favipiravir pharmacokinetics was described by a one-compartment model with an enzyme-dependent elimination(2) (Eq. 1-4 in Supplementary methods).” Line 135-137

Magnitude and decay were obtained by applying the study protocol (Line 59) to those models.

**- Why do you assume the interferon renders target cells refractory rather than blocking infection and production as modeled in influenza?**

Our main assumption was that interferon renders target cells refractory to infection, as we found in Ebola virus infection in NHP (4). Per your comment, we now test other alternative models for the immune response proposed in other acute viral infections. We tested models where F reduces viral infectivity or decreases the rate of viral production (5), increases the viral clearance or the loss rate of infected cells (6). We rewrote the sections methods and results accordingly. Selection was made on a statistical criterion (BIC), and our main result remains unchanged, with the refractory model providing the best fit to the data. It is noteworthy that this prediction is in line with the immune response during infection with Lassa virus *in vitro* and *in vivo*, where type-I interferon production was associated with a control of viral replication (7)(8).

The paper was modified as follows

[**Methods] line 178-194**

“We considered other modes of action of the immune response, using the same parametrization that was used in previous works. We tested several scenarios where F either acted by reducing viral infectivity, decreasing the rate of viral production, increasing the viral clearance or the loss rate of infected cells through a cytotoxic effect (Supplementary equations S5-8).”

**[Results] Line 276-283**

**Challenging immune response's mode of action**

“The refractory model outperformed all other models of immune response, with BIC difference of more than 9 points (S2 Table).”

Supplementary table S2:

| **Immune response** | **Cytotoxicity** | **Blocking infection** | **Blocking production** | **Increasing viral clearance** | **Refractory** |
| --- | --- | --- | --- | --- | --- |
| **BIC** | 511.45 | 499.89 | 506.95 | 506.20 | 490.70 |

[**Discussion] Line 432-449**

Here our model focused primarily on the innate immune response. Following previous findings in Ebola virus (9), our model assumed an effect of the immune response in rendering cells refractory to infection, that could reflect the effects of IFN$\alpha$. We also considered other models where an immune compartment could reduce viral infectivity, viral production, or increase the loss rate of free viruses and infected cells (Supplementary equations S5-9), but all models showed deteriorated statistical criterions in terms of BIC. Our prediction of the importance of IFN$\alpha$ is in line with in vitro findings (7) and in NHP (8). Finally, and unlike what was done in Ebola infections, we did not incorporate an adaptive immune response in the model. Although IgG and IgM may be detectable 15 days after infection (8), it is noteworthy that the viral load concentrations did not show a rapid decline after peak viral load. Even surviving animals with undetectable viral titers at all times had detectable viral load until 40 days after infection, suggesting a modest role of the adaptive immune response in viral clearance.”

**- Are you looking at four or five models? Define them better and separate the results.**

We rewrote and clarified model building per your previous comment.

**- It is not clear which parameters are fixed and which are fitted to the data. It is not until table 2 is referenced that one can infer it.**

We added a specific paragraph entitled “Assumptions on fixed parameter values”. We also added in Table 2 the value of all fixed parameters to ensure easy reproducibility.

**- Are you fitting censored data?**

The information provided by censored data, i.e., data below the lower limit of quantification, is taken into account in the likelihood formulation using the methodology developed in (10, 11). We added the references line 168:

“Information brought by data under limit of quantification was taken into account in parameter estimation (10, 11).”

**- Explain your assumption regarding data fitting. For example: say you are using the first or second version of equation (10). What do you mean by deteriorated fitting criterion? What do you mean by 'at odds with data'. Show what you get or not get. Quantify it. Talk goodness of fit.**

We have rewritten the section on data fitting per your comment to clarify the mechanism of action of drugs lines 153, 174, 255 and 257:

$\frac{dV_{ni}}{dt}=p (1-\mu) (1-\epsilon_{drug}) I_{2} - cV_{ni}$ if treatment blocks production

$\frac{dV_{ni}}{dt}=p (1-(\mu(1-\epsilon_{drug}))) I_{2} - cV_{ni}$if treatment makes virus non-infectious

The fitting criterion used throughout our analyses is the Bayesian Information Criterion (BIC), as added in methods line 169: “Statistical criterion used for model discrimination was the BIC.”

In the results, we now discus qualitatively and quantitatively the differences between the two models line 263:

“Assuming that both drugs block viral production not only deteriorated data fitting criterion (i.e. increased BIC by 10 points) but also provided inconsistent predictions. Indeed, the model could not reproduce the observation of a lower ratio of infectious virus in NHP treated animals (Fig 2, S4 Fig.a). In contrast, assuming an effect in reducing the proportion of infectious virus could recapitulate this pattern, that was in addition predicted to be favipiravir concentration-dependent (S4 Fig.b).”

Moreover, we added a figure comparing the individual estimates of $\mu$in the two models. While the model assuming an effect in viral infectivity provided similar distribution across groups, the model assuming an effect in viral production led to estimates of µ that were lower in treated animals compared to controls, showing the lack of consistency of this model.

We added this element into the results Line 271-272:

“Further, the analysis of the individual parameter estimates showed differences across the treatment groups in the distribution of the proportion of infectious virus, μ. This is at odds with the fact that μ is not related to treatment, and therefore should be similarly distributed in all groups”

**- Can you really fit both Theta and Phi?**

The parameter $\phi$, which quantified the rate at which target cells are made refractory to the infection, was precisely estimated, with a 95% CI equal to 0.31-0.54 (Table 2).

We nonetheless agree that the estimation of Theta was not as precise, as shown by the large 95% CI: 0-393.7 (Table 2). The relatively poor precision of Theta does not mean that the parameter is not identifiable. To address this question, we considered two alternative models given by:

$$\frac{dT}{dt}=-\beta V_{i}T$$

$$\frac{dT}{dt}=-\beta V_{i}T-\phi T$$

Corresponding to $\theta$ to 0 (Eq. 9b) to ∞ (Eq 9a). As can be seen the two models showed significantly deteriorated fitting criterions, showing that the parameterization used is identifiable, even though Theta is associated with a large uncertainty.

Supplementary table S3:

| **Immune response** | **Refractory with density dependent term** | **No immune response** | **Refractory without F compartment** |
| --- | --- | --- | --- |
| **BIC** | 490.70 | 507.33 | 496.63 |

Per your comment, we now present and discuss the limitation on Theta:

**Results lines 279-283**

“Moreover, we verified the necessity of this immune response as well as the effector compartment (Supplementary equations S9a-b). Those models showed deteriorated statistical criteria in terms of BIC, with increase of at least 6 points, supporting the use of a refractory response with an effector compartment”

**Discussion Lines 435-441**

“We […] verified the necessity of this immune response as well the presence of an effector compartment (Supplementary equations 9a-b), but all alternative models showed deteriorated statistical criteria.”

**- Remove the subtitle in the discussion.**

Done.

**- In the discussion you compare your results to those from Ebola where favipiravir increased virus diversity. You did not look into that scenario in Lassa, so you cannot make that comparison.**
Thank you for raising that point. We agree with your point and we modified the discussion as follows (lines 356-374):

“Another interesting observation was the difference in the drug mechanism of action in the two viral infections. Here, the data showed that favipiravir, and ribavirin to a lesser extent, had an effect in reducing the virus infectivity, as measured by the ratio of TCID_50_ to RNA copies (Fig 2). In the context of Ebola and Marburg infection NHP models, favipiravir was associated with an increased virus diversity (12, 13). A small effect on virus infectivity was reported in (12), but this was not observed in our previous study. Further studies on genomic viruses will be needed to confirm that the strong effect of favipiravir on virus infectivity is also caused by an increased in virus mutagenesis and, if so, what levels of mutagenesis might be associated with “error catastrophe”. In our model, the concentration-effect relationship of favipiravir was associated with a high sigmoidicity, suggesting that error catastrophe can only be observed when the drug concentrations passes a critical threshold. Of note, mutagenesis may also increase the risk of emergence of mutations conferring resistance. Resistance to treatment were not observed in NHP models of Ebola and Marburg infections, but patterns of resistance to resistance to favipiravir were identified in in vitro models of influenza infection (14).

Another important finding of our study was the demonstration that ribavirin had an antiviral efficacy in vivo against Lassa infection. Here again the effect passed through a modulation of the infectivity rather than the viral production. In a previous mice model of Lassa infection, we identified an effect of ribavirin in reducing the loss rate of infected cells but no specific antiviral effect (15). Interestingly and consistent with an effect of ribavirin that would not be purely antiviral, animals treated with ribavirin had a median survival of 20.5 days, as compared to 10 days in animals left untreated or receiving 150 mg/kg/day favipiravir. “

Reviewer #2: In this article, the authors develop viral dynamics models and analyze data from non-human primates to assess the efficacy and mode of action of the drugs favipiravir and ribavirin against Lassa fever. They assume that the drugs could either prevent viral replication or render the virions produced non-infectious, based on previous reports on the modes of action of these drugs. They analyze data from 24 primates administered either favipiravir or ribavirin post challenge and compare with a no treatment group. They also compare their results with independent observations on Ebola virus infection, for which favipiravir was used as treatment. Importantly, the measurements include total viral load as well as infectious virus titre, allowing estimation of the effect of the drugs on the infectiousness of the virus. The model also incorporates the overall drug pharmacokinetics based on the dosing protocols employed. Mixed effects analysis is used for fitting and parameter estimation. The authors find that favipiravir and ribavirin both work against Lassa fever by rendering the virions non-infectious and in a dose dependent manner. Further, favipiravir does so better than ribavirin and better than it performed against Ebola. They predict based on these findings the favipiravir dosage that would be suitable for treatment of Lassa fever in humans.

Overall, the study is carefully done. The results are important. The modeling approach is built on previous formalisms and demonstrating its applicability to in vivo data for Lassa fever for the first time and the ability to make predictions of the suitable dosing in humans make it valuable. The paper is well written. I have a few comments for the authors to consider.

Comments:
1. **The authors explicitly incorporate an innate immune response in the model, which protects cells from getting infected. While this effect is expected in most viral infection settings, it is rarely used explicitly in models. The authors should thus explain in some detail why they chose to do so. It may also help to state explicitly what components of the innate immune response are important in the present setting. NK cells and interferon, for instance, could have opposite effects on the outcomes of infection (e.g., see** [**https://www.pnas.org/content/116/35/17393**](https://www.pnas.org/content/116/35/17393)**)**

We thank you for raising this point that was also pointed by the other reviewer.

Our main assumption was that interferon renders target cells refractory to infection, as we found in Ebola virus infection in NHP (4). Per your comment, we now test other alternative models for the immune response proposed in other acute viral infections. We tested models where F reduces viral infectivity or decreases the rate of viral production (5), increases the viral clearance or the loss rate of infected cells (6). We rewrote the sections methods and results accordingly. Selection was made on a statistical criterion (BIC), and our main result remains unchanged, with the refractory model providing the best fit to the data. It is noteworthy that this prediction is in line with the immune response during infection with Lassa virus *in vitro* and *in vivo*, where type-I interferon production was associated with a control of viral replication (7)(8).

The paper was modified as follows

[**Methods] line 178-194**

“We considered other modes of action of the immune response, using the same parametrization that was used in previous works. We tested several scenarios where F either acted by reducing viral infectivity, decreasing the rate of viral production, increasing the viral clearance or the loss rate of infected cells through a cytotoxic effect (Supplementary equations S5-8).”

**[Results] Line 276-283**

**Challenging immune response's mode of action**

“The refractory model outperformed all other models of immune response, with BIC difference of more than 9 points (S2 Table).”

[**Discussion] Line 432-449**

Here our model focused primarily on the innate immune response. Following previous findings in Ebola virus (9), our model assumed an effect of the immune response in rendering cells refractory to infection, that could reflect the effects of IFN$\alpha$. We also considered other models where an immune compartment could reduce viral infectivity, viral production, or increase the loss rate of free viruses and infected cells (Supplementary equations S5-9), but all models showed deteriorated statistical criterions in terms of BIC. Our prediction of the importance of IFN$\alpha$ is in line with in vitro findings (7) and in NHP (8). Finally, and unlike what was done in Ebola infections, we did not incorporate an adaptive immune response in the model. Although IgG and IgM may be detectable 15 days after infection (8), it is noteworthy that the viral load concentrations did not show a rapid decline after peak viral load. Even surviving animals with undetectable viral titers at all times had detectable viral load until 40 days after infection, suggesting a modest role of the adaptive immune response in viral clearance.”

**2. The authors argue that the timing of the start of treatment is crucial for achieving viral clearance. Does this have to do with the viral load rising with time and thus treatment having to suppress a much larger viral pool when started late or is it something more fundamental that is changing with time, like the innate immune response? In other words, can I think of outcomes of treatment being dependent on the viral load at the start of treatment regardless of when the treatment is initiated or does the treatment initiation time have an explicit role? Could the authors clarify and explain? Also, if the timing is really the issue, how could the time of infection be estimated?**

The timing of treatment is crucial to protect the pool of susceptible cells and prevent peak viral load and possibly exacerbated inflammatory response. By initiating treatment before peak, one reduces the exponential growth rate of virus and give more time for the immune response to mount and put more target cells in a refractory state (4, 16–18). Pertaining to the clinical implications of these results, the challenge is that patients may arrive at the hospital after the peak viral load. For instance in the PALM study (19), most Ebola infected patients initiated treatment 4 to 6 days after symptom onset while the peak is 4-5 days after symptom onset (20).

We clarified this in the discussion section: “As discussed by we and others, antiviral treatment needs to be initiated before most target cells have been infected (i.e., before peak viral load) to have a large efficacy (4, 16–18). By reducing peak viral load, treatment will in addition reduce the antigen stimulation and reduce the risk of a dysregulated immune responses. In clinical setting, the implication of these findings is treatment should be initiated as early as possible (21) and before peak viral load.“ Lines 392-399

**3. Lethal mutagenesis and error catastrophe have a threshold like dependence on the mutation rate. As a first approximation, the error threshold is ~1/L, where L is the length of the viral genome (see the work of Manfred Eigen, Peter Schuster and colleagues). The authors do argue that of the two dosages of favipiravir used, the lower does not trigger an error catastrophe and the higher does. Are there in vitro estimates of the mutation rate induced by favipiravir as a function of its concentration that they could use to justify this claim at least based on the ~1/L value? If not, could they propose further studies that would be needed to establish the error threshold for Lassa virus and the associated favipiravir dosage? There seem to be few examples of mutagenic drugs inducing error catastrophe in vivo. It would be remarkable therefore if the authors could demonstrate this. This may also have clinical implications. Because of the threshold behavior, suboptimal dosing may lead to an increase in the mutation rate and hence viral diversity that may favor immune escape or trigger drug resistance. Ensuring that the threshold is crossed may thus be important.**

We thank the reviewer for this important question. We have added the following in the discussion (see also our response to reviewer 1)

*“*Another interesting observation was the difference in the drug mechanism of action in the two viral infections. Here, the data showed that favipiravir, and ribavirin to a lesser extent, had an effect in reducing the virus infectivity, as measured by the ratio of TCID_50_ to RNA copies (Fig 2). In the context of Ebola and Marburg infection NHP models, favipiravir was associated with an increased virus diversity (12, 13). A small effect on virus infectivity was reported in (12), but this was not observed in our previous study. Further studies on genomic viruses will be needed to confirm that the strong effect of favipiravir on virus infectivity is also caused by an increased in virus mutagenesis and, if so, what levels of mutagenesis might be associated with “error catastrophe”. In our model, the concentration-effect relationship of favipiravir was associated with a high sigmoidicity, suggesting that error catastrophe can only be observed when the drug concentrations passes a critical threshold. Of note, mutagenesis may also increase the risk of emergence of mutations conferring resistance. Resistance to treatment were not observed in NHP models of Ebola and Marburg infections, but patterns of resistance to resistance to favipiravir were identified in in vitro models of influenza infection (14).” Lines 356-374

**Minor comments:
1. Line 90: Should I2 be I¬1?**

The typo has been corrected.

**2. In Eqs 7 and 8, the Hill coefficient is set to 1 for ribavirin but a much higher value for favipiravir. Is this just for simplicity or is there evidence of the coefficient being 1 for ribavirin?**

Ribavirin hill coefficient could not be identified due to the fact that only one dose was used in the study. This has been clarified in the manuscript “We did not consider a Hill coefficient different than 1 since only one dose was used.” Line 151

**3. In Eqs. 9 and 10, it may help to distinguish between the two modes of drug action using different symbols or subscripts. Right now, they are both epsilon.**

Treatment effect was clarified using subscript FPV or RBV, and “drug” when it was common to both drugs. Line 144

**4. Line 150: The viral clearance rates in tissues and plasma are set to the same value, saying that the same has been done with Zika and Ebola. It may help to add a line saying why this works with Zika and Ebola.**

We apologize for the confusion we caused by using this wording. We rephrased this section to:

“Virion clearance in both plasma, noted c, respectively, was fixed to 20 d^-1^, similar to what has been performed in Zika and Ebola viruses (4, 22). For simplification, we fixed virion clearance $c_{t}$ in the tissues to the same value.” Line 155-162

**5. Line 191: Ribavirin concentration is assumed fixed. Ribavirin concentration, however, is known to show massive accumulation with time, which the authors too recognize in their pharmacokinetics model. Should this pharmacokinetics not be used for predictions, especially since the dosing for Lassa fever may not last longer than the timescale of the concentration build up?**

We used a PK model for ribavirin for our analyses, however the goal of our prediction was not to determine a dosing regimen for ribavirin, but a clinically coherent concentration associated with efficacy (here the median concentration found using the current treatment protocol). Moreover, human pharmacokinetics at this dosing regimen are unknown, as the doses are much larger than in other infections. Therefore, this would be a limitation to translating these results to man.

**6. Should drug resistance be a cause of worry at all? Is it reported? Perhaps a comment about it in the discussion would help.**

We thank the reviewer for raising this question. Only *in vitro* resistance has been reported in an artificially produced mutant grown as a clone (14), not i*n vivo* neither in Ebola or Influenza A.

We added in the discussion: “Of note, mutagenesis may also increase the risk of emergence of mutations conferring resistance. Resistance to treatment were not observed in NHP models of Ebola and Marburg infections, but patterns of resistance to resistance to favipiravir were identified in in vitro models of influenza infection.” Lines 371-374

**7. In Fig. 1, there seems to be a long tail with the low favipiravir dosage? Is that just a sampling issue or is it systematic?**

Indeed, 3 animals of the low dosage favipiravir died within 10 days while one animal survived until day 20. This is not a sampling issue, as the vital status was collected every day, and just reflects natural variability.

**References**

1.	K. Best, A. S. Perelson, Mathematical modeling of within-host Zika virus dynamics. *Immunol. Rev.* **285**, 81–96 (2018).

2.	A. Vincent Madelain, *et al.*, Favipiravir Pharmacokinetics in Nonhuman Primates and Insights for Future Efficacy Studies of Hemorrhagic. **61**, 1–12 (2017).

3.	C. Lin, L. Yeh, T. Luu, D. Lourenco, J. Y. N. L. Johnson, Pharmacokinetics and Metabolism of [ 14 C ] Ribavirin in Rats and Cynomolgus Monkeys. **47**, 1395–1398 (2003).

4.	V. Madelain, *et al.*, Ebola viral dynamics in nonhuman primates provides insights into virus immuno-pathogenesis and antiviral strategies. *Nat. Commun.* **9**, 1–11 (2018).

5.	A. M. Smith, A. S. Perelson, Influenza A virus infection kinetics: quantitative data and models. *WIREs Syst. Biol. Med.* **3**, 429–445 (2011).

6.	S. Koyama, K. J. Ishii, C. Coban, S. Akira, Innate immune response to viral infection. *Cytokine* **43**, 336–341 (2008).

7.	S. Baize, *et al.*, Role of interferons in the control of Lassa virus replication in human dendritic cells and macrophages. *Microbes Infect.* **8**, 1194–1202 (2006).

8.	S. Baize, *et al.*, Early and Strong Immune Responses Are Associated with Control of Viral Replication and Recovery in Lassa Virus-Infected Cynomolgus Monkeys ᰔ. **83**, 5890–5903 (2009).

9.	V. Madelain, *et al.*, Ebola viral dynamics in nonhuman primates provides insights into virus immuno-pathogenesis and antiviral strategies. *Nat. Commun.* **9**, 4013 (2018).

10.	A. Samson, M. Lavielle, F. Mentré, Extension of the SAEM algorithm to left-censored data in nonlinear mixed-effects model: Application to HIV dynamics model. *Comput. Stat. Data Anal.* **51**, 1562–1574 (2006).

11.	R. Thiébaut, *et al.*, Estimation of dynamical model parameters taking into account undetectable marker values. *BMC Med. Res. Methodol.* **6**, 38 (2006).

12.	N. Espy, *et al.*, T-705 induces lethal mutagenesis in Ebola and Marburg populations in macaques. *Antiviral Res.* **170**, 104529 (2019).

13.	J. Guedj, *et al.*, Antiviral efficacy of favipiravir against Ebola virus: A translational study in cynomolgus macaques. *PLoS Med.* **15**, 1–21 (2018).

14.	D. H. Goldhill, *et al.*, The mechanism of resistance to favipiravir in influenza. *Proc. Natl. Acad. Sci. U. S. A.* **115**, 11613–11618 (2018).

15.	P. Carrillo-Bustamante, *et al.*, Determining Ribavirin’s mechanism of action against Lassa virus infection. *Sci. Rep.* **7**, 1–12 (2017).

16.	L. E. Friberg, J. Guedj, Acute bacterial or viral infection—What’s the difference? A perspective from PKPD modellers. *Clin. Microbiol. Infect.* (2020) https:/doi.org/10.1016/j.cmi.2019.12.008.

17.	A. Gonçalves, *et al.*, Timing of Antiviral Treatment Initiation is Critical to Reduce SARS-CoV-2 Viral Load. *CPT Pharmacometrics Syst. Pharmacol.* **n/a** (2020).

18.	C. Hadjichrysanthou, *et al.*, Understanding the within-host dynamics of influenza A virus: From theory to clinical implications. *J. R. Soc. Interface* **13** (2016).

19.	S. Mulangu, *et al.*, A Randomized, Controlled Trial of Ebola Virus Disease Therapeutics. *N. Engl. J. Med.* **381**, 2293–2303 (2019).

20.	F. O., *et al.*, Use of Viremia to Evaluate the Baseline Case Fatality Ratio of Ebola Virus Disease and Inform Treatment Studies: A Retrospective Cohort Study. *PLoS Med.* **12** (2015).

21.	P. Okokhere, *et al.*, Clinical and laboratory predictors of Lassa fever outcome in a dedicated treatment facility in Nigeria: a retrospective, observational cohort study. *Lancet. Infect. Dis.* **18**, 684–695 (2018).

22.	K. Best, *et al.*, Zika plasma viral dynamics in nonhuman primates provides insights into early infection and antiviral strategies. *Proc. Natl. Acad. Sci.* **114**, 8847–8852 (2017).
